# A Hilbert Transform-Based Smart Sensor for Detection, Classification, and Quantification of Power Quality Disturbances

**DOI:** 10.3390/s130505507

**Published:** 2013-04-25

**Authors:** David Granados-Lieberman, Martin Valtierra-Rodriguez, Luis A. Morales-Hernandez, Rene J. Romero-Troncoso, Roque A. Osornio-Rios

**Affiliations:** 1 HSPdigital-CA Mecatronica, Facultad de Ingenieria, Universidad Autonoma de Queretaro, Campus San Juan del Rio, Rio Moctezuma 249, Col. San Cayetano, San Juan del Rio, Qro. 76807, Mexico; E-Mails: dgranados@hspdigital.org (D.G.-L.); mvaltierra@hspdigital.org (M.V.-R.); luis.morales@uaq.mx (L.A.M.-H); 2 HSPdigital-CA Telematica, DICIS, Universidad de Guanajuato, Carr. Salamanca-Valle km 3.5 + 1.8, Palo Blanco, Salamanca, Gto. 36885, Mexico; E-Mail: troncoso@hspdigital.org

**Keywords:** Hilbert transform, power quality disturbances, power quality indices, instantaneous exponential time constant, FPGA, feed forward neural network, smart sensor

## Abstract

Power quality disturbance (PQD) monitoring has become an important issue due to the growing number of disturbing loads connected to the power line and to the susceptibility of certain loads to their presence. In any real power system, there are multiple sources of several disturbances which can have different magnitudes and appear at different times. In order to avoid equipment damage and estimate the damage severity, they have to be detected, classified, and quantified. In this work, a smart sensor for detection, classification, and quantification of PQD is proposed. First, the Hilbert transform (HT) is used as detection technique; then, the classification of the envelope of a PQD obtained through HT is carried out by a feed forward neural network (FFNN). Finally, the root mean square voltage (*Vrms*), peak voltage (*Vpeak*), crest factor (*CF*), and total harmonic distortion (*THD*) indices calculated through HT and Parseval's theorem as well as an instantaneous exponential time constant quantify the PQD according to the disturbance presented. The aforementioned methodology is processed online using digital hardware signal processing based on field programmable gate array (FPGA). Besides, the proposed smart sensor performance is validated and tested through synthetic signals and under real operating conditions, respectively.

## 1. Introduction

Over the past few years, the power quality (PQ) has become an important issue in industrial and academic fields due to the growing number of disturbing loads in the industrial and public sectors; another important factor is the susceptibility that certain loads present to the presence of these disturbances. These anomalies are generally called power quality disturbances (PQD), which are deviations of voltage or current from the ideal sinusoidal waveform, such as sags, swells, interruptions, harmonics, flicker, notching, spikes, and oscillatory transients [1]. In any real power system, there are multiple sources of disturbances which can have different magnitudes and appear at different times. Therefore, and in order to reduce the generated problems caused by PQD, it is necessary to have systems that are able to detect, classify, and quantify automatically the different PQD [2]. This also allows developing solutions for avoiding damage to equipment, extend its lifetime, and reduce costs as well as for estimating the damage severity in the equipment.

At present, different techniques have been used for analyzing PQD, such as short-time Fourier transform (STFT), wavelet transform (WT), S-transform, Kalman filter, Gabor-Wigner, Hilbert transform, and Hilbert Huang transform [3–13]. For instance, STFT gives time–frequency information related to disturbance waveforms [3], but transient signals cannot be adequately described with this methodology due to its fixed window size. To overcome the drawbacks of STFT, the WT provides the time-scale analysis of the non-stationary signal since it decomposes the signal into a time-scale representation rather than a time-frequency representation. Different WT analyses [4], wavelet multiresolution analysis (MRA) [5], and MRA with entropy norm (EN) [6] have been used to detect and classify several types of PQD. Unfortunately, in real practice the WT capabilities are often significantly degraded in noisy environments [7]. For this reason, other schemes based on S-transform [7–9], Kalman filter [10], and Gabor-Wigner transform [11] have been developed for detecting effectively PQD in noisy environments. On the other hand, the Hilbert transform (HT) envelope detection technique [12] and a combination with ensemble empirical mode decomposition (EEMD) called Hilbert Huang transform (HHT) [13] have also been used in PQD monitoring. Unfortunately, none of the aforementioned works provide any information about the PQD quantification which is very important in industrial applications since with this information it is possible to estimate the damage severity in the equipment due to the PQD. Therefore, an online system that detects, classifies, and quantifies the different PQD is a necessity. It is worth noting that in the current literature different systems cover topics concerning online PQD detection and classification; nevertheless, just a few works provide any information related to the characteristics of the different disturbances, such is the case of Radil *et al.* [14], who provided information about time localization, duration, and magnitude of the disturbances using digital filtering, mathematical morphology, root mean square (*RMS*), and peak values; however, more specific characteristics according to the classified PQD are not given. On the other hand, other works propose to obtain several electric power characteristics using PQI. For instance, Mindykoswki *et al.* [15], using techniques such as WT, FFT, and chirp z-transform (CZT) developed a PQ instrument for PQ assessment in ship systems, yet, the classification stage is not carried out; therefore, specific features of each PQD are not obtained.

From the technological and industrial points of view, smart sensors which utilize a standard sensor and includes in their functionalities signal processing, communication, and integration capabilities can be used to overcome the issues of PQ monitoring systems. The term “smart sensor” is employed according to the functionality classification given by Rivera *et al.* [16], from the definitions of the Institute of Electrical and Electronics Engineers [17,18]. On the other hand, smart sensors based on field-programmable gate arrays (FPGA) are capable of performing the task in real time due to their high-speed processing capabilities, configurability, and system-on-a-chip (SoC) solutions for industrial applications [16]. Smart sensors related to PQ monitoring have being applied in different ways [19–22]. For instance, Granados-Lieberman *et al.* [19] developed an FPGA-based smart sensor for real-time high-resolution frequency measurement in accordance with international power quality monitoring standards. Humin *et al.* [20] presented a smart sensor for medium-voltage dc power grid protection *via* current and voltage transformers. On the other hand, a design of wireless sensor networks for a PQ monitoring system in order to customize the distribution pattern of the power quality information is proposed in [21]. Furthermore, Lim *et al.* [22] presented a reliable data delivery mechanism by employing the neural network concept for monitoring basic electrical quantities. Moreover, a sensor to detect a very low direct current (DC) voltage component superimposed onto an alternating current (AC) voltage component is presented in [23]. Due to their proven reliability in other reported tasks of PQ monitoring, smart sensors are suitable candidates for simultaneously detecting, classifying, and quantifying the PQD in a SoC solution, rather than having different systems for each task of detecting, classifying, and quantifying PQD.

The contribution of this work is the development of a smart sensor for online detection, classification, and quantification of single PQD. Another contribution of this work is the proposed methodology due to its simplicity and to the theoretical foundation by depending largely on the HT. First, the HT is used as detection technique; then, a feed-forward neural network (FFNN) performs the classification of the PQD envelope provided by the HT. Finally, the root mean square voltage (*Vrms*), peak voltage (*Vpeak*), crest factor (*CF*), and total harmonic distortion (*THD*) indices calculated through the HT and Parseval's theorem, as well as an instantaneous exponential time constant, are used for quantifying the PQD according to the disturbance presented. All the aforementioned methodology is implemented into an FPGA for a SoC solution thanks to its high-performance computational capabilities for industrial and online applications. Besides, the proposed smart sensor performance is validated and tested using synthetic signals and under real operating conditions, respectively.

## 2. Theoretical Background

### 2.1. Power Quality Disturbances

The power quality indices (PQI), such as *Vrms, Vpeak, CF*, and *THD* are used for illustrating the undesirable impact of electrical disturbances in concordance with the required standards. The electrical disturbances are abnormalities in voltage or current that present variations in magnitude with respect to its nominal value during an interval time. Depending on the thresholds of these features, the IEEE Standard 1159 [24] and the European Standard EN 50160 [25] categorize these disturbances as shown in Table 1. For instance, a sag condition is considered when the *Vrms* value is within the range from 0.1 to 0.9 per unit (pu) of the nominal voltage and has a minimum duration of 0.5 cycles of fundamental frequency. Regarding to the PQD and PQI, the sags, swells, and interruptions are changes in *Vrms*; when these changes are continuous and occur within proper frequency ranges they create the visual phenomenon called flicker [1]. The harmonic distortions are normally estimated through the *THD* and *CF*. On the other hand, voltage notches and spikes are characterized by their amplitude and duration in combination with the point on the sine wave at which the notching starts. In the same way, the oscillatory transients are described by their maximum peak-value or *Vpeak* and exponential time constant [1].

### 2.2. Hilbert Transform

The HT is a mathematical tool used for tracking the voltage envelope [12,26], which is defined for real signals as Equation (1), with its equivalent Fourier transform (FT) version given in Equation (2):

(1) $$x_{HT}(t)=x(t)*\frac{1}{\pi t}$$

(2) $$X_{\text{HT}}(\Omega )=-j\mathrm{sgn}(\Omega )X(\Omega )$$

where −*j sgn*(Ω), has the effect of shifting the negative frequency components of *x*(*t*) by +90° and the positive frequencies components by −90°.

A useful way to understand and to compute the HT of *x*(*t*) is using the analytic signal *z*(*t*) composed by the real signal and the HT shifted 90°, defined as:

(3) $$z(t)=x(t)+jx_{HT}(t)=A(t)e^{j\theta (t)}$$

where *A*(*t*) is called the envelope signal of *x*(*t*) and *θ*(*t*) is called the instantaneous phase signal of *x*(*t*). In terms of *x*(*t*) and *x_HT_*(*t*), it is clear that:

(4) $$A(t)=\sqrt{x^{2}(t)+x_{HT}^{2}(t)}$$

(5) $$\theta (t)=\tan^{-1}\left(\frac{x_{HT}(t)}{x(t)}\right)$$

**Figure 1.**
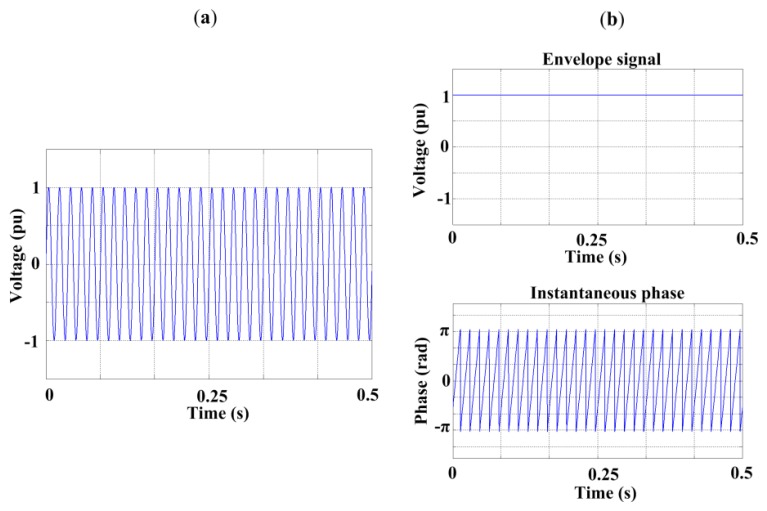
Hilbert Transform. (**a**) Sinusoidal waveform; (**b**) Hilbert Transform results.

The sinusoidal waveform shown in Figure 1(a) has HT envelope and instantaneous phase as shown in Figure 1(b).

### 2.3. Feed Forward Neural Network

**Figure 2.**
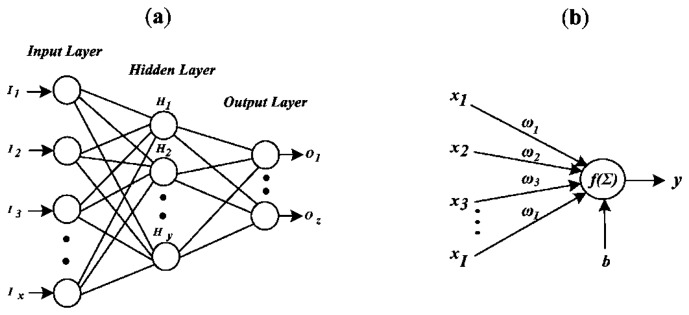
Feed-forward neural network. (**a**) Architecture; (**b**) Neuron.

A FFNN is characterized as having a layered architecture with single or multiple neurons in each layer, as shown in Figure 2(a). In this architecture, the input information moves in one direction only, from the input nodes, through the hidden nodes, and to the output nodes. For characterizing the network weights, pairs of input-output data are presented; then, a training rule for adjusting these weights is used. With that, the error between the desired and calculated outputs is minimized. Finally, the entire training data is repeatedly presented to the FFNN until the overall error is acceptable [27]. On the other hand, the mathematical function that describes to each neuron shown in Figure 2(b) is given in Equation (6); it consists on the summation Σ(·) of the multiplications between the inputs *x_i_* and the associated multipliers commonly called weights ω_i_ to each input plus a bias *b*; then, this result is evaluated with a nonlinear function *f*(·) to provide the FFNN with the ability to model nonlinear relationships [27]. This is applicable to all neurons.

(6) $$y=f\left(\sum_{i=1}^{I}\omega_{i}x_{i}+b\right)$$

## 3. Smart Sensor

**Figure 3.**
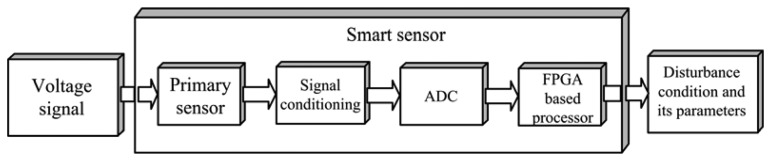
Block diagram of the PQD smart sensor.

In this section, the proposed smart sensor and the algorithm implemented in the FPGA-based processor for detecting, classifying, and quantifying the PQD are described. The proposed smart sensor block diagram is shown in Figure 3.

In order to acquire the voltage signal and get a result, the smart sensor uses firstly a voltage divider with a measurement range from 0 V to 440 V as a primary sensor; the voltage divider arrays are made up of 1 W 120 kΩ and 1 W 3.3 kΩ metal-film resistors. Then, the voltage signal passes through the signal conditioning stage, which contains a precision isolation amplifier model ISO124PND [28] to get galvanic isolation between the power system and the proposed smart sensor, a DCV011515DP DC-DC converter model [29] in order to decouple system references, and an anti-aliasing filter of second-order low-pass Butterworth filter with a cutoff frequency of 3 kHz, allowing the correct analysis of harmonics and transient disturbances with frequencies lower than the cutoff frequency.

Afterwards, the analog-to-digital converter (ADC), which corresponds to a 16-bit 4-channel serial-output ADS8341 [30], using only one channel gives the signal to the FPGA-based processor to determine the disturbance condition and its quantification parameters of a single-phase power system.

**Figure 4.**
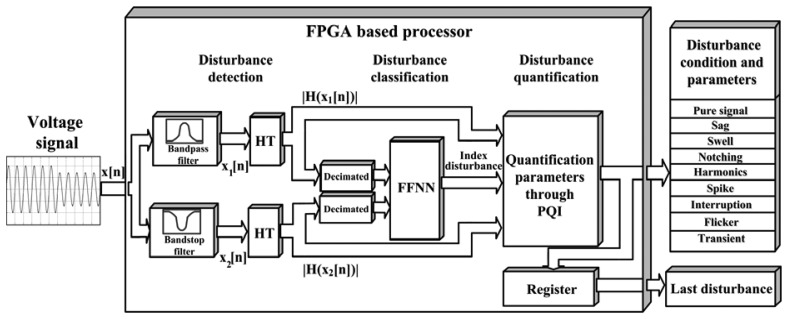
FPGA-based processor.

The architecture of the FPGA-based processor for a single-phase is shown as a block diagram in Figure 4, which can be replicated for three-phase or poly-phase power systems. It is worth noticing that in this work just one phase of the power system is analyzed. The proposed methodology is divided into the detection, classification, and quantification of the voltage signal *x[n]*.

### 3.1. Disturbance Detection

**Figure 5.**
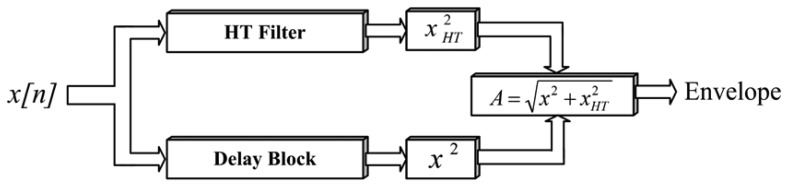
Hilbert transform tracking of the voltage envelope.

For the detection stage, the voltage signal *x[n]* is separated by two digital filters into *x_1_[n]* and *x_2_[n]* which correspond to the fundamental frequency component and the remaining frequency components, respectively, with the objective of separating and detecting the PQD that appear in the fundamental and in the remaining frequencies. The signal *x_1_[n]* is obtained with an order 16 finite impulse response (FIR) Gaussian window filter for a center frequency according to the power system frequency, in this case 60 Hz as fundamental component. On the other hand, the signal *x_2_[n]* is extracted through a second-order infinite impulse response (IIR) notch filter which removes the fundamental frequency component. Then, each HT block of Figure 4 computes Equation (4) according to Figure 5 to extract the envelope signal; there, the HT filter block implements the HT as a FIR linear phase filter of order 32 which is designed through Parks-McClellan method by means of the frequency components shifting by satisfying Equation (2). In a parallel way, the input *x* is delayed by the Delay Block for compensating the sample delay produced by the HT filter; then, the outputs are arithmetically squared, added, and root squared to get the envelope or instantaneous amplitude *A*(*n*).

Both HT blocks shown in Figure 4 are implemented as shown in Figure 5 for *x_1_[n]* and *x_2_[n]*, corresponding to the instantaneous amplitudes *|H(x_1_[n])|* and *|H(x_2_[n])|*, respectively. The disturbance detection is triggered when any change in the signals *|H(x_1_[n])|* and *|H(x_2_[n])|* happens. Once any disturbance in the power line is detected, the signal is classified by means of the FFNN block.

### 3.2. Disturbance Classification

**Figure 6.**
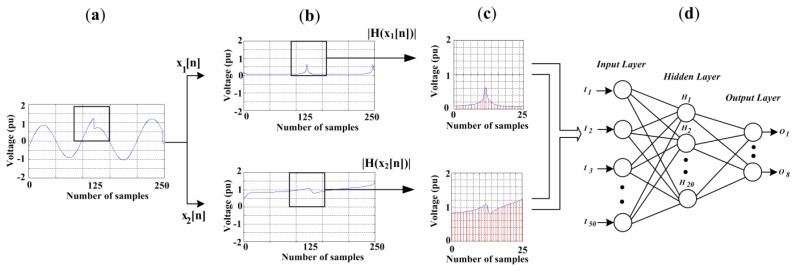
Procedure of classification. (**a**) Sinusoidal wave with spikes; (**b**) HT outputs; (**c**) Decimated HT outputs; (**d**) Proposed FFNN.

The classification stage is carried out by an FFNN, which analyses the envelope signals *|H(x_1_[n])|* and *|H(x_2_[n])|* each half cycle in order to classify the different disturbances. This time window is a running window with size equal to a half period in order to satisfy the minimum duration of a sag, swell or interruption. In order to better explain the classification procedure, Figure 6 is presented. Figure 6(a) shows a sinusoidal wave with spikes, it is sampled at 6,000 Hz which corresponds to 50 samples per half cycle. Then, this signal is passed through the HT blocks to give the signals *|H(x_1_[n])|* and *|H(x_2_[n])|*, as shown Figure 6(b). In order to reduce the dimensionality of the input data some reduction techniques have been reported [31,32]; in this work, for simplicity and without affecting the signal characteristics, the HT outputs are just decimated by 2; thus, the samples number per half cycle is 25 (Figure 6(c)). These samples that make up the PQD waveform are the inputs to the FFNN which has 50 inputs, 20 neurons in the hidden layer, and eight outputs (Figure 6(d)). The eight outputs are one per each disturbance (sag, swell, interruption, harmonic, flicker, notching, spike, and oscillatory transient) since each neuron is set at one if the disturbance exists and to zero when there is no disturbance, yet, it is well known that the FFNN output is rarely one or zero; therefore, a threshold of 0.5 is also used to force the output to one or zero, respectively. Once the PQD is classified, its respective quantification parameters are computed.

### 3.3. Disturbance Quantification

For quantifying the different PQD in the power line, the *Vrms, THD, Vpeak*, and *CF* indices are used. It is convenient to mention that they are the most commonly indices to evaluate the PQ [24,25]. In this work, the mathematical expressions proposed for computing the PQI are founded on the Parseval's theorem for their direct evaluation through the HT.

The *RMS* value or effective value of the discrete HT *H[n]* can be obtained as follows:

(7) $$\text{RMS}=\sqrt{\frac{\overset{L}{\underset{n=1}{\text{∑}}}H^{2}[n]}{2L}}$$

where *L* is the samples number of the analyzed time window.

On the other hand, the *Vpeak* corresponds to the maximum value of the signal in the analyzed interval; therefore, it is the maximum value of the summation of the instantaneous values of *|H(x_1_[n])|* and *|H(x_2_[n])|* according to Equation (8) in a time window. Likewise, the *Vpeak* of *|H(x_2_[n])|* shown in Equation (9) allows quantifying short duration disturbance as spikes, notching or oscillatory transients:

(8) $$\text{Vpeak}=\max\left(\left|H\left(x_{1}[n]\right)\right|+\left|H\left(x_{2}[n]\right)\right|\right)$$

(9) $$\text{Vpeak}\_H_{2}=\max\left(\left|H\left(x_{2}[n]\right)\right|\right)$$

Another important PQI is the *THD*, which is a parameter defined as the *RMS* value of the harmonic content divided by the *RMS* value of the fundamental component, usually multiplied by 100 for a percentage result [1]. The *THD* is obtained with the *RMS* values of the decomposed signals according to Equation (10):

(10) $$\text{THD}=\frac{\text{RMS}\_H_{2}}{\text{RMS}\_H_{1}}\cdot 100\%$$

where *RMS_H*_1_ and *RMS_H*_2_ are the *RMS* values of the signals *|H(x_1_[n])|* and *|H(x_2_[n])|*, respectively.

In the same way, the *CF* is a time-domain property that indicates how much distortion has the top of the sine wave and it is given by Equation (11):

(11) $$CF=\frac{\text{Vpeak}}{\text{RMS}\_H}$$

where *RMS_H* is equal to the sum of *RMS_H*_1_ and *RMS_H*_2_.

For quantifying and mainly knowing the exponential time constant in the oscillatory transients, the following mathematical expressions are used. First, the mathematical expression for modeling an oscillatory transient in a sinusoidal wave is:

(12) $$V[n]=A\sin\left(2\pi fn\right)+Be^{-\tau \left(n-N\right)}\sin\left(2\pi f_{1}(n-N)\right)$$

where *A* is amplitude of the nominal voltage, *f* is the frequency power system, *f*_1_ is the transient frequency, *B* is the amplitude of the transient, *N* is the number of shifted samples where the transient starts, *τ* is the disturbance exponential time constant, and *n* = 0, 1, 2,..L is the actual sample. Then, the proposed methodology separates the two terms of Equation (12) by means of the two filters; thus, the *|H(x_2_[n])|* obtained after the HT block corresponds to the envelope of the second term in Equation (12). Therefore and by considering *N* = 0, the *|H(x_2_[n])|* amplitude is directly related with the exponential term as follows:

(13) $$|Hx{\text{x}}_{2}\left[\text{n}])\right|=Be^{-\tau \cdot n}$$

In order to compute *τ*, Equation (13) is differentiated as shown in Equation (14) and arranged in Equation (15) which allow computing an instantaneous *τ*:

(14) $${\left|H({\text{x}}_{2}\left[\text{n}]\right)\right|}^{\prime }=-\tau Be^{-\tau \cdot n}$$

(15) $$\tau =-\frac{{|H({\text{x}}_{2}\left[\text{n}])\right|}^{\prime }}{|H({\text{x}}_{2}\left[\text{n}])\right|}$$

For improving the computation of *τ*, the evaluation of the derivative in the actual sample *n* according to Equation (15) is obtained through an averaging discrete-difference filter as follows:

(16) $$\tau =-\frac{|H({\text{x}}_{2}[\text{n}-2])|-|H({\text{x}}_{2}\left[\text{n}])\right|}{|H({\text{x}}_{2}[\text{n}-1])|}$$

In short, Table 2 shows the kinds of disturbances analyzed by the smart sensor as well as a description of the quantification parameters of each disturbance such as magnitude *M*, period of notching and spike *T*, *Vpeak*, *THD*, flicker period *T_FL_*, flicker magnitude *M_FL_*, exponential time constant or mean lifetime *τ*. Regarding the flicker, its quantification parameters are related only with the tracking of voltage flicker; however, they can be used in others systems for correlating other variables such as the eye response to flicker perception of lamps or statistical measures of short and long-terms flicker severity. It is worth noticing that the disturbance duration *Δt* is also given by the smart sensor when the disturbance has finished. Finally, the proposed smart sensor has a register block at the output, as shown Figure 4, which stores the quantified parameters of the last disturbance occurred.

## 4. Experimentation and Results

In this section, the validation and the experimental setup for evaluating the performance of the proposed smart sensor are presented.

### 4.1. Training and Validation Stages

**Figure 7.**
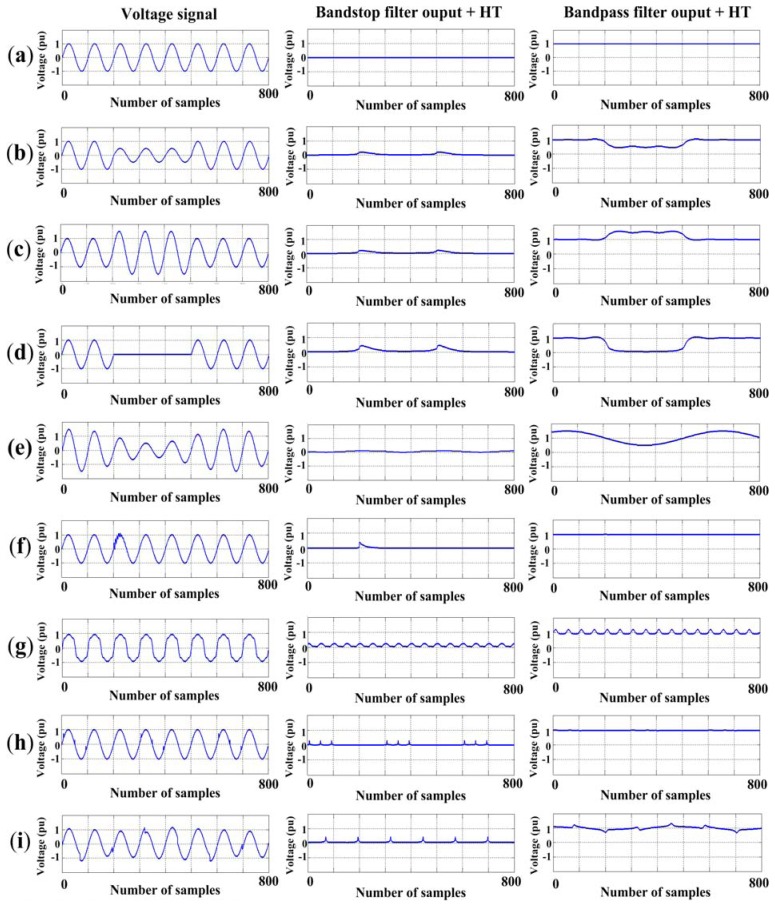
PQD generated. (**a**) Pure signal; (**b**) Sag; (**c**) Swell; (**d**) Interruption; (**e**) Flicker; (**f**) Transients; (**g**) Harmonics; (**h**) Notching; (**i**) Spikes.

In order to validate the proposed methodology, it has been tested with synthetic signals to have *a priori* knowledge of the true PQD values and thus, the difference or error between the true value and the obtained value can be estimated. Firstly, a database with 200 signals is built for each one of the eight PQD, plus 200 for pure signals; these signals are generated in concordance with the equations and the parameters variation shown in Table 3, some of them have been used in [6] and [9],whereas the others are proposed in this research. Figure 7 shows a signal of each disturbance generated as well as its respective behavior through the filters and HT. For each PQD, the 200 signals are divided into 100 for training and 100 for validating the proposed methodology, respectively.

Regarding the NN structure and training, a log-sigmoid activation function into the overall FFNN is used, the training goal is set at 10^−6^, and the training rule is the Levenberg-Marquardt algorithm; all the aforementioned is carried out offline using the MATLAB software; once the NN is built, trained, and validated their coefficients (weights and biases) are used into digital structures that computes Equation (6) as shown in [33]. In both the training and validation stages, the synthetic signals are processed by the two filters, HT, quantification, and FFNN blocks. The overall methodology is implemented and validated using the MATLAB software. Since noise is present in all electrical power distribution networks, the proposed methodology is also tested in a noisy environment by adding Gaussian noise with a level of −20 dB using the noiseless signal as reference.

The obtained results of the overall methodology are separated into two tables. Table 4 shows the percentage of effectiveness for detecting and classifying PQD; on the other hand, Table 5 shows the quantification results. Regarding Table 4, the first column indicates the kind of PQD, the columns two and three indicate the percentages of effectiveness in noiseless and noisy conditions, respectively. For instance, the light-gray row in Table 4 that reads sag, 100, and 100 for noiseless and noisy conditions, respectively, means that the methodology accurately classifies all signals used in the validation stage. On the other hand, the quantification effectiveness of the proposed methodology is estimated through the mean squared error (MSE) which quantifies the difference between each value obtained by an estimator (the proposed methodology) and the real value for each signal as follows:

(17) $$\text{MSE}=\frac{1}{n}\sum_{I=1}^{n}{\left({\hat{y}}_{i}-y_{i}\right)}^{2}$$

where *ŷ* is the obtained value, *y* is the real value, and *n* is the number of signals. Table 5 shows the MSE results for each disturbance and its respective quantification parameters, as an example, the light-gray row in Table 5 depicts the quantification MSE under noiseless and noisy conditions, being the noiseless condition 0.1036, and the noisy condition 1.3833; being the last condition thirteen times higher than the former.

### 4.2. Experimental Setup

**Figure 8.**
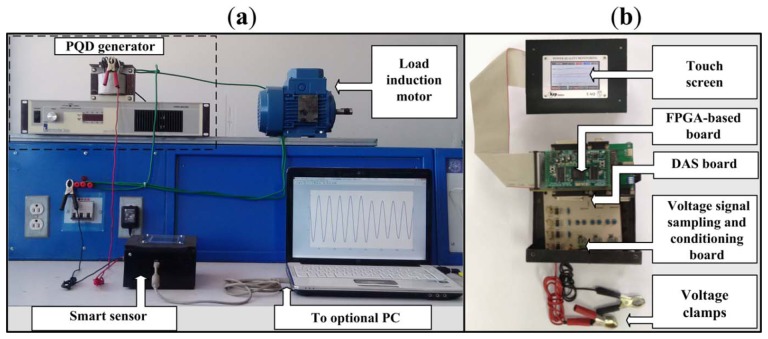
Smart sensor under real operating conditions. (**a**) Experimental setup; (**b**) Smart Sensor.

On the other hand, the proposed methodology implemented on the smart sensor is also tested under real operating conditions according to the experimental setup shown in Figure 8(a); the developed smart sensor is shown in Figure 8(b), which is implemented in a proprietary Spartan 3E XC3S1600 FPGA platform [34] running at 48 MHz; Table 6 summarizes the resource usage of the FPGA.

The experimental setup consists of an electric load (induction motor) of 1-hp (746 W) which is fed by a three-phase power electric system. Then, a proprietary PQD digital generator injects the disturbance to one phase, monitored by the smart sensor. In Table 7 the column for the generated PQD shows the kind of disturbance and its quantification parameters, as well as its used reference values, taking 20 runs of each PQD condition, showing as result the mean (μ), standard deviation (σ) and mean error. For instance, the light-gray row reads a sag condition with a real magnitude of 115.8, μ = 115.7942, σ = 0.0633, and an error of 0.0057 volts. In order to estimate the accuracy and precision of the smart sensor, a Fluke 435-II was used as reference. The accuracy and precision for voltage-related parameters are estimated with Equations (18) and (19), respectively, as well as with the values of the pure signal in Table 7.

Therefore, the smart-sensor accuracy is estimated to be 99.84% with a precision of 99.95%:

(18) $$\text{Accuracy}(\%)=100-\frac{\left(V_{\text{ref}}-V_{\text{mean}}\right)}{V_{\text{ref}}}\cdot 100$$

(19) $$\text{Precision}(\%)=100-\frac{\sigma }{V_{\text{ref}}}\cdot 100$$

where *V_ref_* is the voltage reference value, *V_mean_* is the voltage mean value obtained of the measurements set, and σ is its standard deviation.

### 4.3. Real Signals

**Figure 9.**
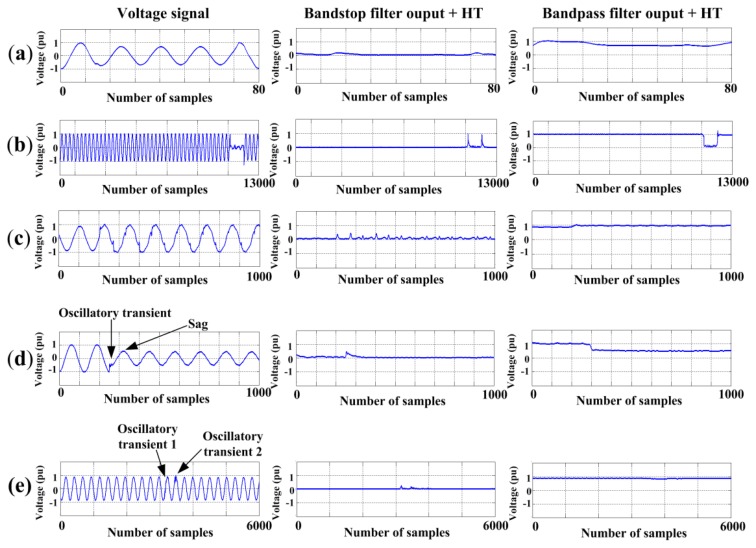
Real signals of PQD. (**a**) Sag (overhead insulator failure); (**b**) Interruption (overloaded transformer); (**c**) Spikes (splice failure on the aerial cable); (**d**) Oscillatory transient and sag (terminator failure on the cable dip); (**e**) Two oscillatory transients (cable fault on the underground portion).

To evaluate the performance of the proposed approach for real-world measurements, 40 real measurements of PQD from IEEE work group (P1159.3) are analyzed. Figure 9 shows just some real signals analyzed for different PQD as well as their respective behavior through the filters and HT. On the other hand, Table 8 presents in normalized way (according to the nominal voltage value) the quantification parameters obtained by the proposed approach for the signals shown in Figure 9. Regarding Figure 9(d,e), they show a signals with two PQD which occur at different times, their parameters of quantification are also presented in Table 8 by considering that the proposed approach first gives the parameters for the first disturbance and then the second one according to the detected PQD.

### 4.4. Analysis and Discussions

From Table 4, it can be seen that the majority of PQD conditions are classified correctly. The worst classification errors occur for the harmonics condition under noiseless and noisy conditions; yet, the classification effectiveness is over 97% and 89%, respectively. This decrease in the classification effectiveness is due to the fact that the FFNN classifies the waveform and as it is well known the different combinations of harmonics constitute different waveforms. The best classification results (100%) are for pure signal, sag, swell, and interruption since the general waveform of these PQD rarely changes. On the other hand, the effectiveness of classification shown in Table 5 for noiseless and noisy condition through MSE indicates a high accuracy in all tests, since an MSE of almost a relative zero means that the smart sensor obtains results very similar to the real ones. In addition, the MSE in noisy conditions is almost ten times higher than in noiseless condition, as expected by adding 20 dB of Gaussian noise. The highest errors obtained by the smart sensor are for the calculation of τ since it is computed through a derivative and although it is used an averaging discrete-difference filter its susceptibility to noise generate small variations in the result; yet, its mean error is below 0.2% according to the obtained results shown in Table 7.

The detection and classification tasks are critical in PQ monitoring. First, the disturbance must be detected in order to be classified. Then, an accurate classification of the disturbance is necessary to assert that the computed quantification parameters are appropriate. In this work and regarding the real signals, the correctly detection and classification of the proposed approach are demonstrated with the results shown in Figure 9 and Table 8, even when there are two immediate disturbances as shown in Figures 9(d,e). Therefore, if the detection and classification are performed well the quantification parameters for the different disturbances are computed correctly.

On the other hand, Table 9 shows a comparison of the main characteristics between the reported works in the literature and the one here proposed. Regarding the hardware implementation, most works are personal computer (PC)-based, which can compromise the online operation, and only this work and reference [15] present a SoC solution, yet the proposed smart sensor has a classification stage unlike the solution proposed in [15]; besides, it has the option to send the data for PC post-processing as done in other reported works and systems. The detection of PQD is already reported in [4,11]; however, their methodologies do not embrace a classification stage. On the contrary, the works [5,6,8] report different techniques for PQD classification, yet, the noisy condition is not considered. Unlike the aforementioned works, the noisy condition in [7,9,10,12,13] is considered. It must be noticed that only this work presents quantification as PQI and also as specific disturbance-related parameters such as time delay constant of an oscillatory transient, the period and magnitude of a notching or flicker, and so on, whereas other works [14,15] present results as PQI only.

## 5. Conclusions

This work proposes a new smart sensor for online detection, classification, and quantification of PQD using only a voltage divider as a primary sensor, which results in a highly-portable instrument. The overall methodology is based on HT; first, the detection is carried out when there is any change in the HT envelope of a voltage's nominal signal. Then, the FFNN classifies the waveform given by the HT in a half cycle. Finally, the PQI computed through the HT and Parseval's theorem quantify the disturbance. All the aforementioned demonstrate the capabilities of HT as a powerful tool of easy implementation through filters for the detection, classification, and quantification of PQD. On the other hand, the obtained smart sensor results under synthetic and real operating conditions show its accuracy, precision, and immunity to noisy environments being evident its industrial applicability. Besides, the fact that the smart sensor develops the three tasks for PQ analysis makes it more attractive than having different systems or techniques for each task of detecting, classifying, and quantifying PQD unlike other reported works.

The proposed smart sensor is based on FPGA technology which provides high computation performance for online operation of the proposed methodology, as well as a low-cost, portable and efficient SoC solution. This implementation shows that an FPGA platform is a suitable solution for smart processing units in developing smart sensors. On other hand, the proposed methodology, as well as the developed smart sensor, can be utilized for further research development in the area of power quality monitoring by adding control tasks for each PQ disturbance as well as in studies of the PQD repercussion in divers susceptible loads or electric systems. Besides, the smart sensor can be integrated in other systems or instruments for many other applications such as protection systems, data loggers, control systems, and so on. Finally, the proposed methodology can be used as reference to develop other approaches to detect, classify, and quantify combined PQD.

## Figures and Tables

**Table 1.** Power quality disturbances classification.

**PQ Disturbance**	**Duration**	**Values**
Sag	>0.5 cycles	0.1 to 0.9 pu
Swell	>0.5 cycles	1.1 to 1.8 pu
Interruption	>0.5 cycles	<0.1 pu
Flicker	-	0.9 to 1.1 pu
Harmonic	-	*THD* >5%

**Table 2.** Quantification parameters for power quality disturbances.

**Disturbance Condition**	**Parameters**	**Description**
Pure signal	*M* (*V*)	*M* is the *RMS* voltage given in Equation (7) during a time window *Δtw* equal to half cycle of fundamental frequency.
Sag	*M* (*V*), *Δt* (*s*)	*M* is the *RMS* voltage obtained each *Δtw* during the entire sag, swell or interruption, respectively.
Swell	*M* (*V*), *Δt* (*s*)	
Interruption	*M* (*V*), *Δt* (*s*)	
Notching	*T* (*s*),	The period *T* and amplitude *Peak_H*_2_ are estimated by means of a zero crossing and by Equation (9), respectively, each *Δtw*.
	*Peak_H*_2_ (*V*),	
	*Δt* (*s*)	
Spike	*T* (*s*),	
	*Peak_H*_2_ (*V*),	
	*Δt* (*s*)	
Harmonics	*THD* (%), *CF*(-), *Δt* (*s*)	The *THD* and *CF* indices are obtained each *Δtw* according to (10) and (11), respectively.
Flicker	*T_FL_* (*s*),	The period *T_FL_* and amplitude *M_FL_* are estimated by means of a first zero crossing and by Equations (9) and (7), respectively.
	*M_FL_* (*V*),	
	*Δt* (*s*)	
Oscillatory Transient	*Peak_H*_2_ (*V*),	The amplitude *Peak_H*_2_ and the instantaneous exponential time constant τ are computed by Equations (9) and (16), respectively. *Peak_H*_2_ is given once that the transient is done.
	*τ* (-), *Δt* (*s*)	

(-): Dimensionless.

**Table 3.** Power quality disturbances models.

**Power Quality Disturbance**	**Equations**	**Parameters Variation**
Pure signal [6,9]	*v*(*k*) = *A*sin(2*πfk*)	-
Interruption [6,9]	*d*(*k*) = − *αA* (*u*(*k*−*k*_1_) − *u* (*k* − *k*_2_)) sin)2*πfk*)	0.9 ≤ *α* ≤ 1; *k*_1_ <*k*_2_
Sag [6,9]	*d*(*k*) = − *αA* (*u*(*k* − *k*_1_) − *u*(*k* − *k*_2_)) sin(2*πfk*)	0.1 ≤ *α* ≤ 0.9; *k*_1_ <*k*_2_
Swell [6,9]	*d*(*k*) = *αA*(*u*(*k* − *k*_1_) − *u*(*k* − *k*_2_)) sin(2*πfk*)	0.1 ≤ *α* ≤ 0.8; *k*_1_ <*k*_2_
Harmonics [6,9]	$d(k)=\overset{M}{\underset{m=1}{\text{∑}}}A_{m}\sin(2\pi h_{m}fk)$	M: total number of harmonics 0.05≤ *A_m_* ≤ 0.5, 2≤ *h_m_* ≤ 40
Oscillatory Transients [6,9]	*d*(*k*) = *βe*^−*γ*(*k*−*k*_1_)^ sin(2 *πfk*)	-5 ≤ *β* ≤ 5, 50≤ *γ* ≤ 100
Flicker [6,9]	*d* (*k*) = *αA* sin(2 *πf_r_k*)sin(2*πfk*)	1 ≤ *f_r_* ≤ 10, 0 < *α* ≤ 0.2
Notching (P)	*d*(*k*) = *α*⌊ |sin(2 *πβk*) | ⌋	60 < *β* ≤ 240, 0 < *α* ≤ 0.2
Spikes (P)	*d*(*k*) = *α* ⌊ | sin(2*πβk*)| ⌋	1< *β* ≤ 10, 0.1≤ *α* ≤ 0.5

*u* (·): step function; ⌊·⌋: floor function; |·|: absolute value; (P): Proposed.

**Table 4.** Proposed methodology effectiveness for detection and classification in noiseless (NL) and noisy (N) conditions.

**Power Quality Disturbance**	**Percentage of Effectiveness for Detection and Classification Stages (%)**	

	**NL**	**N**
Pure signal	100	99
Interruption	100	100
Sag	100	100
Swell	100	100
Harmonics	97	89
Oscillatory Transients	99	93
Flicker	98	92
Notching	98	91
Spikes	98	90

**Table 5.** MSE for quantification of PQD in noiseless (NL) and noisy (N) conditions.

**Power Quality Disturbance**	**Parameters**	**Units**	**MSE**	

			**NL**	**N**
Pure signal	*M*	*Volt RMS*	0.1492	1.7339
Interruption	*M*	*Volt RMS*	0.0002	0.0022
	*Δt*	*s*	1.0746e–8	15.9184e–8
Sag	*M*	*Volt RMS*	0.1036	1.3833
	*Δt*	*s*	0.2637e–6	2.9531e–6
Swell	*M*	*Volt RMS*	0.1496	1.5578
	*Δt*	*s*	0.2312e–6	2.9380e–6
Harmonics	*THD*	%	0.0003	0.0029
	*CF*	-	21.7617e–6	253.8832e–6
	*Δt*	*s*	7.8870e–6	99.8392e–6
Oscillatory Transients	*Peak_H*_2_	*Volt peak*	0.0014	0.0163
	*τ*	-	0.7682	9.8955
	*Δt*	*s*	1.4705e–10	18.4283e–10
Flicker	*T_FL_*	*s*	0.0844e–6	0.8132e–6
	*M_FL_*	*Volt RMS*	0.1398	1.6017
	*Δt*	*s*	8.2753e–6	92.4175e–6
Notching	*T*	*s*	2.9290e−10	40.5973e–10
	*Peak_H*_2_	*Volt peak*	0.0003	0.0037
	*Δt*	*s*	0.2369e–6	3.1097e–6
Spikes	*T*	*s*	6.2842e–10	60.8252e–10
	*Peak_H*_2_	*Volt peak*	0.0020	0.0264
	*Δt*	*s*	0.2136e–6	3.2049e–6

**Table 6.** Resource usage of the FPGA.

**Resource Utilization**	**Xilinx Spartan 3E XC3S1600E**
Slices	9440/14,752 (64%)
Slice flip flops	15047/29,504 (51%)
4-input LUTs	8605/29,504 (29%)
Maximum operation frequency	54.127 MHz

**Table 7.** Smart sensor performance under real operating conditions.

**Generated Power Quality Disturbances**				**Smart Sensor Results**		

Condition	Parameters	Reference values	Units	Mean (μ)	Standard deviation (σ)	Error (real-μ)
Pure signal	*M*	127.2	*Volt RMS*	127.0021	0.0605	0.1979
Interruption	*M*	0	*Volt RMS*	0.0035	0.0524	−0.0035
	*Δt*	0.0333	*s*	0.0323	0.0059	0.0010
Sag	*M*	115.8	*Volt RMS*	115.7943	0.0633	0.0057
	*Δt*	0.1666	*s*	0.1680	0.0051	−0.0014
Swell	*M*	138.2	*Volt RMS*	138.1995	0.0685	0.0005
	*Δt*	0.1666	*s*	0.1671	0.0062	−0.0005
Harmonics	*THD*	6	%	5.9873	0.0480	0.0127
	*CF*	1.2	-	1.2049	0.0519	−0.0049
	*Δt*	1	*s*	0.9897	0.0548	0.0103
Oscillatory Transients	*Peak_H*_2_	12.72	*Volt peak*	12.7160	0.0608	0.0040
	*τ*	300	-	300.5209	0.0594	−0.5209
	*Δt*	0.0042	*s*	0.0040	0.0051	0.0002
Flicker	*T_FL_*	0.1	*s*	0.1084	0.0508	−0.0084
	*M_FL_*	133.56	*Volt RMS*	133.5469	0.1151	0.0131
	*Δt*	1	*s*	1.0111	0.0565	−0.0111
Notching	*T*	0.0055	*s*	0.0052	0.0022	0.0003
	*Peak_H*_2_	6.36	*Volt peak*	6.3815	0.0493	−0.0215
	*Δt*	0.1666	*s*	0.1639	0.0175	0.0027
Spikes	*T*	0.0083	*s*	0.0086	0.0024	−0.0003
	*Peak_H*_2_	15.26	*Volt peak*	15.2807	0.0652	−0.0207
	*Δt*	0.1666	*s*	0.1695	0.0174	−0.0029

**Table 8.** Classification and quantification results of the proposed approach under real signals.

**Real Signals of PQD**	**PQD Classification**	**PQD Quantification**	

		**Parameters**	**Results**
(a) Overhead insulator failure.	Sag	*M*	0.71193
		*Δt*	0.05833
(b) Overloaded transformer.	Interruption	*M*	0.08594
		*Δt*	0.05816
(c) Splice failure on the aerial cable.	Spikes	*T*	0.00861
		*Peak_H*_2_	0.30352
		*Δt*	0.09911
(d) Terminator failure on the cable dip.	Oscillatory transient	*Peak_H*_2_	0.37721
		*τ*	250.741
		*Δt*	0.00759
	Sag	*M*	0.54571
		*Δt*	0.09189
(e) Cable fault on the underground portion.	Oscillatory transient 1	*Peak_H*_2_	0.25708
		*τ*	307.485
		*Δt*	0.00673
	Oscillatory transient 1	*Peak_H*_2_	0.19113
		*τ*	917.251
		*Δt*	0.00274

**Table 9.** Main characteristics of previous works and of the proposed work.

		**Hardware**		**Noise Condition**		**Capabilities**		**Results**	
	
**Work**	**Used Technique**	**PCBased**	**SoC Based**	**Noiseless**	**Noisy**	**Detect**	**Classify**	**PQI**	**Disturbance-Related Parameters**
[4]	Wavelet	X		X		X			
[5]	MRA	X		X		X	X		
[6]	MRA with EN	X		X		X	X		
[7]	S-transform	X		X	X	X	X		
[8]	S-transform	X		X		X	X		
[9]	S-transform	X		X	X	X	X		
[10]	Kalman filter	X		X	X	X	X		
[11]	Gabor–Wigner Transform	X		X		X			
[12]	HT	X		X	X	X	X		
[13]	HHT	X		X	X	X	X		
[14]	Mathematical morphology	X		X	X	X	X	X	
[15]	WT, FFT, CZT	X	X	X	X	X		X	
Proposed	HT	X	X	X	X	X	X	X	X
